# Genome-wide analysis of androgen receptor binding and transcriptomic analysis in mesenchymal subsets during prostate development

**DOI:** 10.1242/dmm.039297

**Published:** 2019-07-25

**Authors:** Claire Nash, Nadia Boufaied, Dunarel Badescu, Yu Chang Wang, Miltiadis Paliouras, Mark Trifiro, Ioannis Ragoussis, Axel A. Thomson

**Affiliations:** 1 Department of Surgery, Division of Urology, McGill University and the Cancer Research Program of the Research Institute of McGill University Health Centre, Montreal, Quebec, Canada H4A 3J1; 2 McGill University and Genome Quebec Innovation Center, Montreal, Quebec, Canada H3A 0G1; 3 Division of Endocrinology, Department of Medicine, Sir Mortimer B. Davis-Jewish General Hospital, 5750 Côte-des-Neiges Rd, Montreal, QC, Canada H3S 1Y9

**Keywords:** ChIP-seq, Androgen receptor, RNA-seq, Single-cell RNA-seq, Mesenchyme, Prostate development

## Abstract

Prostate development is controlled by androgens, the androgen receptor (AR) and mesenchymal–epithelial signalling. We used chromatin immunoprecipitation sequencing (ChIP-seq) to define AR genomic binding in the male and female mesenchyme. Tissue- and single-cell-based transcriptional profiling was used to define mesenchymal AR target genes. We observed significant AR genomic binding in females and a strong enrichment at proximal promoters in both sexes. In males, there was greater AR binding to introns and intergenic regions as well as to classical AR binding motifs. In females, there was increased proximal promoter binding and involvement of cofactors. Comparison of AR-bound genes with transcriptomic data enabled the identification of novel sexually dimorphic AR target genes. We validated the dimorphic expression of AR target genes using published datasets and confirmed regulation by androgens using *ex vivo* organ cultures. AR targets showed variable expression in patients with androgen insensitivity syndrome. We examined AR function at single-cell resolution using single-cell RNA sequencing (scRNA-seq) in male and female mesenchyme. Surprisingly, both AR and target genes were distributed throughout cell subsets, with few positive cells within each subset. AR binding was weakly correlated with target gene expression.

## Abstract (precis)

**Summary:** A study of how androgens lead to sexually dimorphic development of the prostate using transcription factor genome binding and transcriptome analysis in mesenchymal subsets.

## INTRODUCTION

During embryogenesis, the prostate forms through epithelial budding from the urogenital sinus (UGS) in response to testosterone (Cooke et al., 1987; Georgas et al., 2015; Kellokumpu-Lehtinen et al., 1980; Lung and Cunha, 1981); see Toivanen and Shen (2017) for a recent review. Circulating testosterone levels are higher in males than in females because of synthesis by the testes (Siiteri and Wilson, 1974), which leads to sexual dimorphism of the UGS. The importance of testosterone in this process has been demonstrated using *in vivo* and *in vitro* studies, whereby embryonic UGS can be induced to form prostatic structures when stimulated with testosterone (Cunha, 1975; Lasnitzki and Mizuno, 1977; Takeda et al., 1986). Testosterone and the more potent metabolite dihydrotestosterone (DHT) (Berman et al., 1995; Horton, 1992) activate the androgen receptor (AR), which leads to nuclear translocation and regulation of gene transcription. AR signalling is known to be essential for prostate organogenesis as male embryos with mutant AR fail to form prostate glands (de Gendt et al., 2004; Lyon and Hawkes, 1970; Yeh et al., 2002).

Studies in rats have shown that, during initial stages of development, AR expression is restricted to the mesenchymal cells of the UGS (Hayward et al., 1996). The importance of mesenchymal AR signalling has been demonstrated using rodent tissue recombination models. A study using *Tfm* mice, which express a mutant AR, showed that mesenchymal AR is required for prostate induction but epithelial AR alone is not sufficient to drive prostate development (Cunha and Chung, 1981). Prostatic growth is stimulated throughout neonatal growth by testosterone (Donjacour and Cunha, 1993). Epithelial growth, differentiation and budding during prostate development is dependent on mesenchymal–epithelial interactions (Cai et al., 2013; Cooke et al., 1987; Cunha et al., 2002) and may be stimulated through the release of paracrine factors from the mesenchyme in response to androgens (Cunha et al., 1992), although defining the identity of these has been difficult.

The downstream targets of the AR signalling pathway and the molecular mechanisms that drive sexual dimorphism of the prostate are currently poorly defined, because AR signalling and target genes have not been characterized in mesenchymal cells. The andromedin hypothesis suggests that AR signalling in the UGS mesenchyme leads to upregulation of paracrine factors (andromedins) that signal directly to the epithelium to induce growth and budding of the prostate (reviewed in Tenniswood, 1986; Thomson, 2008). Although several fibroblast growth factors (Fgfs) and other molecules have been proposed to act as andromedins, none have comprehensively fulfilled the proposed requirements. Furthermore, studies that used discovery-based approaches to perform an unbiased identification of candidate AR mechanisms did not focus on mesenchyme and yielded variable results, and lack consensus.

To define the genes involved in prostate organogenesis, several studies conducted molecular profiling of whole developing prostate UGS tissues in rodents. These studies profiled whole UGS tissues at a range of developmental timepoints, from E16 continuing through to neonatal and adult timepoints (Abbott et al., 2003; Pritchard et al., 2009; Schaeffer et al., 2008; Zhang et al., 2006). Through comparison of dissociated UGS mesenchyme and epithelium from males and females at various timepoints using LongSAGE, Zhang and colleagues were able to identify sex- and cell-type-specific genes associated with prostate organogenesis (Zhang et al., 2006). These include members of the Wnt, Notch and TGF-β pathways, with secreted molecules such as Sfrp2 enriched in UGS mesenchyme at key developmental timepoints. Many of these genes were also identified as differential between male and female UGS mesenchyme by microarray profiling (Pritchard et al., 2009). One of the limitations of these studies was the use of whole tissues comprised of multiple cell types; such cellular complexity compromises the detection of genes expressed in cell subsets. Prostate mesenchyme has been shown to be comprised of subsets that contain organ inductive activity (Thomson et al., 2002; Timms et al., 1995), and a few studies have addressed transcript expression within these subsets (Boufaied et al., 2017; Vanpoucke et al., 2007).

The role of androgens in gene regulation during prostate development has received relatively little investigation. One study used microarray profiling to identify androgen-induced gene expression changes in female UGS following intrauterine exposure to DHT (Schaeffer et al., 2008). This revealed signalling pathways such as TGF-β, Wnt and Notch as well as epigenetic chromatin remodelling, proliferation and MAPK pathways that were either induced or suppressed by androgen stimulation. However, it was unclear whether the gene expression responses to androgens were a result of direct AR–DNA binding and transcriptional regulation or caused by secondary (indirect) effects of AR signalling. In addition, for the majority of these datasets it is difficult to assign transcriptional changes to specific cell types such as epithelium versus mesenchyme because of the cellular complexity of the whole UGS tissues used for profiling. Studies have shown that in tissue with as little as 15% epithelial cell content, 50% of the resulting transcriptome is epithelial in origin as a result of the increased RNA yield of epithelial cells versus stromal cells (de Bruin et al., 2005). For this reason, it is likely that mesenchymal transcriptional changes are underrepresented in the majority of whole UGS tissue molecular profiling datasets.

We have applied genome-wide binding and transcriptional profiling to identify mesenchyme-derived AR target genes (ARTGs) that are important for sexual dimorphism of the UGS. We used microdissected tissue subsets from both female and male UGS in order to derive mesenchymal-specific ARTGs and identify transcriptional differences between males and females. Using ChIP sequencing (ChIP-seq) of these tissue subsets, we were able to identify potential direct ARTGs. Comparison of these with RNA sequencing profiles of the same tissue allowed us to examine which of these genes were sexually dimorphic between female and male UGS. To address issues of cellular complexity within male UGS tissues, which were comprised of both mesenchyme and epithelium, we derived sexually dimorphic transcripts using single-cell RNA sequencing (scRNA-seq) of ventral prostate (VP) versus cells of a specific area of mesenchyme in female UGS termed the ventral mesenchymal pad (VMP) (Thomson and Cunha, 1999; Timms et al., 1995; Vanpoucke et al., 2007). We validated both sexual dimorphism and response to androgens of a panel of these genes by quantitative real-time PCR (qPCR). We extended the cellular resolution using scRNA-seq data for cell subpopulations and distribution of AR and its target genes, and were able to address AR signalling profiles at the single cell level to gain a better understanding of AR function in mesenchymal tissues of the developing prostate.

## RESULTS

### AR protein expression and ChIP sequencing of microdissected rat tissues

**Fig. 1.**
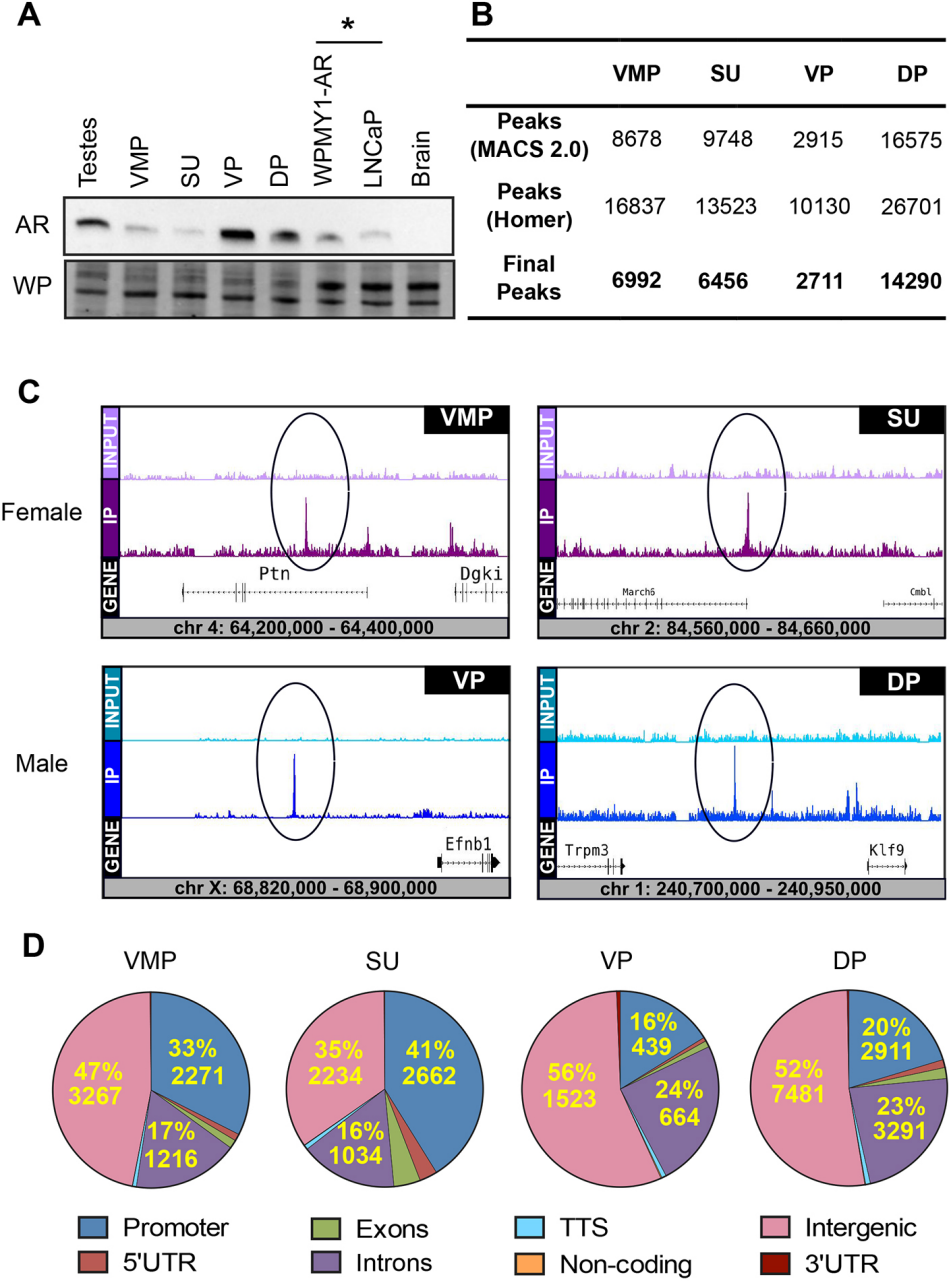
**AR protein expression and genome-wide AR binding in P0 rat urogenital tract tissues.** (A) Western blot analysis of AR (110 kDa); male rat testes tissue, female ventral mesenchymal pad tissue (VMP), female smooth muscle and urethra tissue (SU), male ventral prostate tissue (VP), male dorsal prostate tissue (DP) all at P0, WPMY1-AR prostate fibroblast cell line, LNCaP prostate cancer cell line (positive controls), mixed sex brain tissue at P0 (negative control). Whole protein (WP) was visualized and used as loading control. * indicates lysates diluted 1:100 with male rat brain tissue lysate. Representative blot from two biological replicates and two technical replicates (*n*=4). (B) Overview of AR ChIP-seq peaks of VMP, SU, VP and DP tissues detailing number of peaks called by MACS2.0 and HOMER algorithms and final co-identified peak libraries used in subsequent analyses. (C) Gene track examples of VMP, SU, VP and DP called peaks (IP) vs input controls at loci on chromosomes 4, 2, X and 1, respectively (rn6). (D) Genomic location analysis of AR peaks in VMP, SU, VP and DP in comparison to the whole genome (rn6). AR peaks were enriched 32–41% within the promoter regions of genes (−1000 bp to +100 bp from the transcriptional start site) in female tissues compared with only 16–20% in male tissues. 5′UTR, 5′ untranslated region; 3′UTR, 3′ untranslated region, TTS, translational termination site.

Tissues were microdissected from day of birth (P0) rats to isolate the female ventral mesenchymal pad (VMP), female adjacent urethra (comprised of smooth muscle, peri-urethral stroma and urethral epithelia; SU), male ventral prostate (VP) and male dorsal/dorsolateral prostate (DP). AR protein levels in the four tissues were measured by western blotting using the AR H280 antibody (Fig. 1A and Fig. S1). Rat testes and brain tissue (P0) were included as positive and negative controls for AR, respectively. In addition, WPMY1-AR stromal cells and LNCaP prostate cancer cells were diluted 1:100 in brain extract to serve as positive controls for AR. We observed lower levels of AR protein in female VMP and SU tissues than in male VP and DP tissues. AR protein levels in cell line positive controls were at least 100 times greater than in all four tissue types. To validate the distribution of AR in tissue, immunohistochemistry was performed to confirm western blot data suggesting enriched mesenchymal expression of AR. The VMP is mesenchymal and lacks epithelia, and there is little AR in the urethral epithelia within SU (Fig. S2), indicating that these tissues were good models of mesenchymal AR. VP and DP samples were comprised of mesenchyme and significant epithelial content. Although it was not feasible to further microdissect VP and DP samples to separate epithelia and mesenchyme, there was relatively little AR expression in epithelial cells (Fig. S2) suggesting that most of the AR signal originated in VP and DP mesenchyme. We propose that the four tissue types used for analysis are good models of mesenchymal AR action and target gene identification, albeit with some epithelial contamination in male tissues.

To assess the suitability of these tissues for ChIP sequencing analysis, ChIP was performed on tissue pools of VMP, SU, VP and DP followed by qPCR analysis of a known ARTG *Zbtb16* (Yu et al., 2010) to confirm the efficiency of the ChIP reaction prior to sequencing. We saw enrichment of AR binding events at a genomic site 91 kb downstream of the transcriptional start site (TSS) of *Zbtb16* with about 1.5- to 28-fold enrichment. Male tissues showed a stronger enrichment at this site than female tissues (Fig. S3).

Following ChIP-seq, we observed numerous AR peaks in all samples with both MACS (2.1.0) and HOMER, despite the lower levels of AR in female samples. We defined 6992 peaks in VMP, 6456 peaks in SU, 2711 peaks in VP and 14,290 peaks in DP (Fig. 1B and Table S1). The abundance of peaks in female samples (VMP and SU) suggested that AR protein level did not correlate with the number of AR ChIP-seq peaks, and that low AR levels led to high peak numbers in females. This was consistent with a previous study using human primary prostate stromal cells (Nash et al., 2017), which showed that low AR levels in stromal cells was correlated with high peak numbers. We were able to detect AR peaks in regions of known ARTGs such as *Ptn* (Orr et al., 2011) in female tissues and *Trpm3* in males (Fig. 1C).

We observed differences in the genomic distribution of AR binding between male and female tissues. Female VMP and SU tissues had an unusually high proportion of AR binding at proximal promoter regions of genes; defined as +100 bp to −1000 bp surrounding the TSS. VMP and SU showed 33% (2271) and 41% (2662) of total peaks at gene promoters whereas VP and DP showed 16% (439) and 20% (2911), respectively (Fig. 1D). The enrichment of AR at sites proximal to the TSS is a feature of stromally expressed AR (Nash et al., 2017). In males, we observed a modest enrichment at intronic sites (24%, 23%) compared with females (17%, 16%). These data document the distribution of AR in promoter and intronic sites in males and females and show sexually dimorphic differences in genomic location. The AR showed a surprisingly strong genomic localization in females despite low levels of AR protein.

**Fig. 2.**
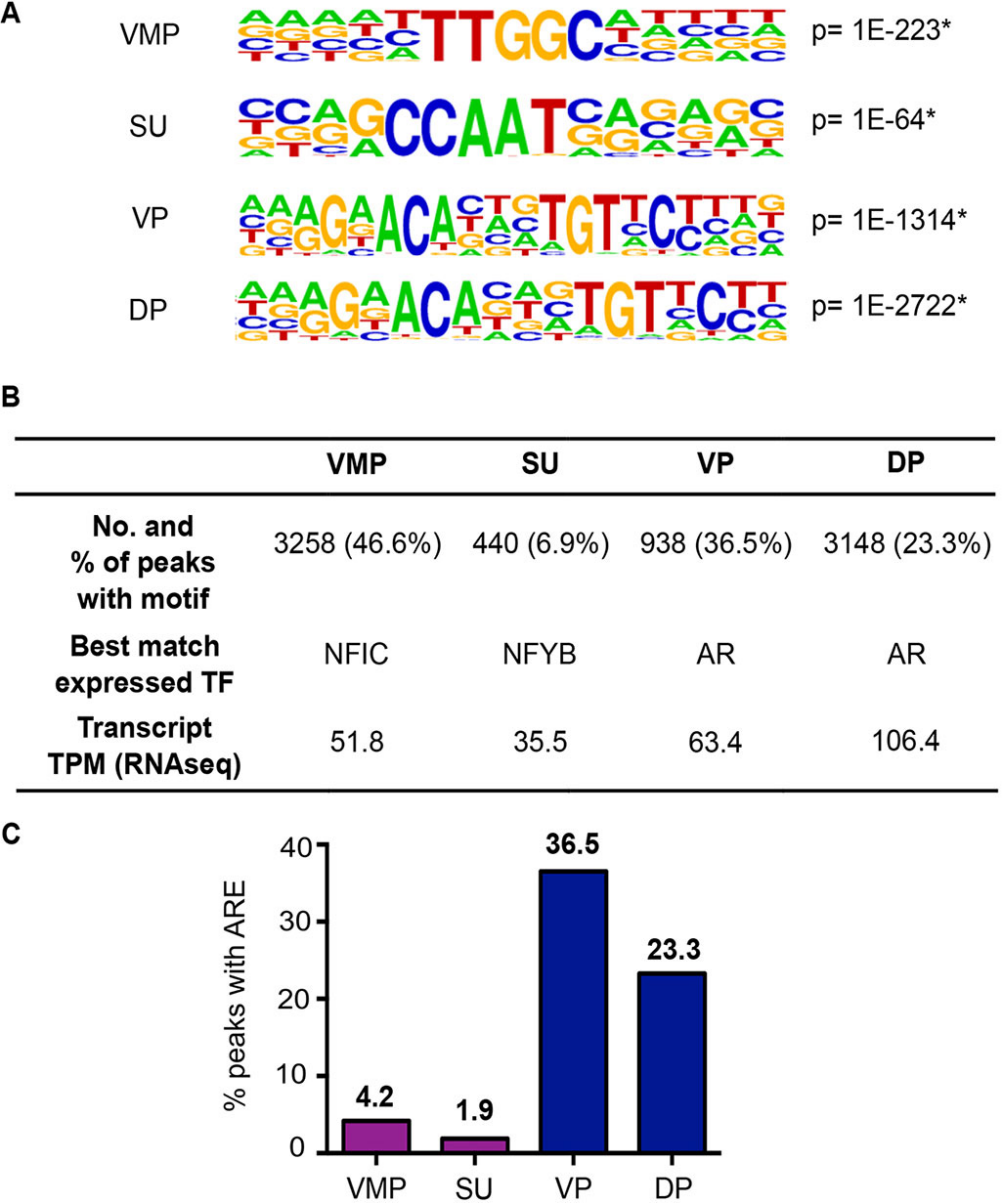
***de novo* sequence motif analysis of AR peaks in P0 rat urogenital tract tissues.** (A) Consensus sequence logos of the top sequence motifs and their significance identified in female VMP and SU tissues and in male VP and DP tissues using *de novo* motif analysis with HOMER. *P<1E-50. (B) An overview of the number of AR peaks containing the top sequence motif, the best match transcription factor (TF) based on positive mRNA expression from RNA-seq analysis and expression value in TPM. (C) Histogram showing the percentage (bold) of AR peaks positive for classical androgen-response elements (ARE). Male VP and DP tissues had approximately ten times more ARE-positive peaks than female VMP and SU tissues.

To characterize AR binding sequences and look for potential AR co-factors in our tissues, we performed *de novo* motif analysis on VMP, SU, VP and DP peaks using HOMER. We observed that AR in female tissues (VMP and SU) did not bind to the classical androgen-response elements (AREs) observed in male tissues (VP and DP) (Fig. 2A). We stratified potential AR binding motifs using transcript expression data (RNA-seq) for the identified co-factor, to restrict our analysis to those co-factors expressed in the tissues. We selected the most probable binding site and then focussed on those factors expressed more than 10 transcripts per million (TPM). This identified *Nfic* and *Nfyb* as probable co-factors for AR in VMP and SU (female) tissues, respectively (Fig. 2B). We also compared the number of ARE-positive peaks in our four tissue samples (Fig. 2C) and in combined female and male peaks (Fig. S4) and observed 9–30 times less ARE peaks in female tissues than males. In addition, the genomic location of ARE-positive peaks in all samples were enriched at intergenic regions (Fig. 2C and Fig. S4). This suggests that the high proportion of AR peaks at proximal promoter sites in female tissues are largely devoid of AREs. A similar observation was reported in a previous study using human primary prostate stromal cells (Nash et al., 2017).

### Identification of sexually dimorphic transcripts using whole tissue RNA sequencing

**Fig. 3.**
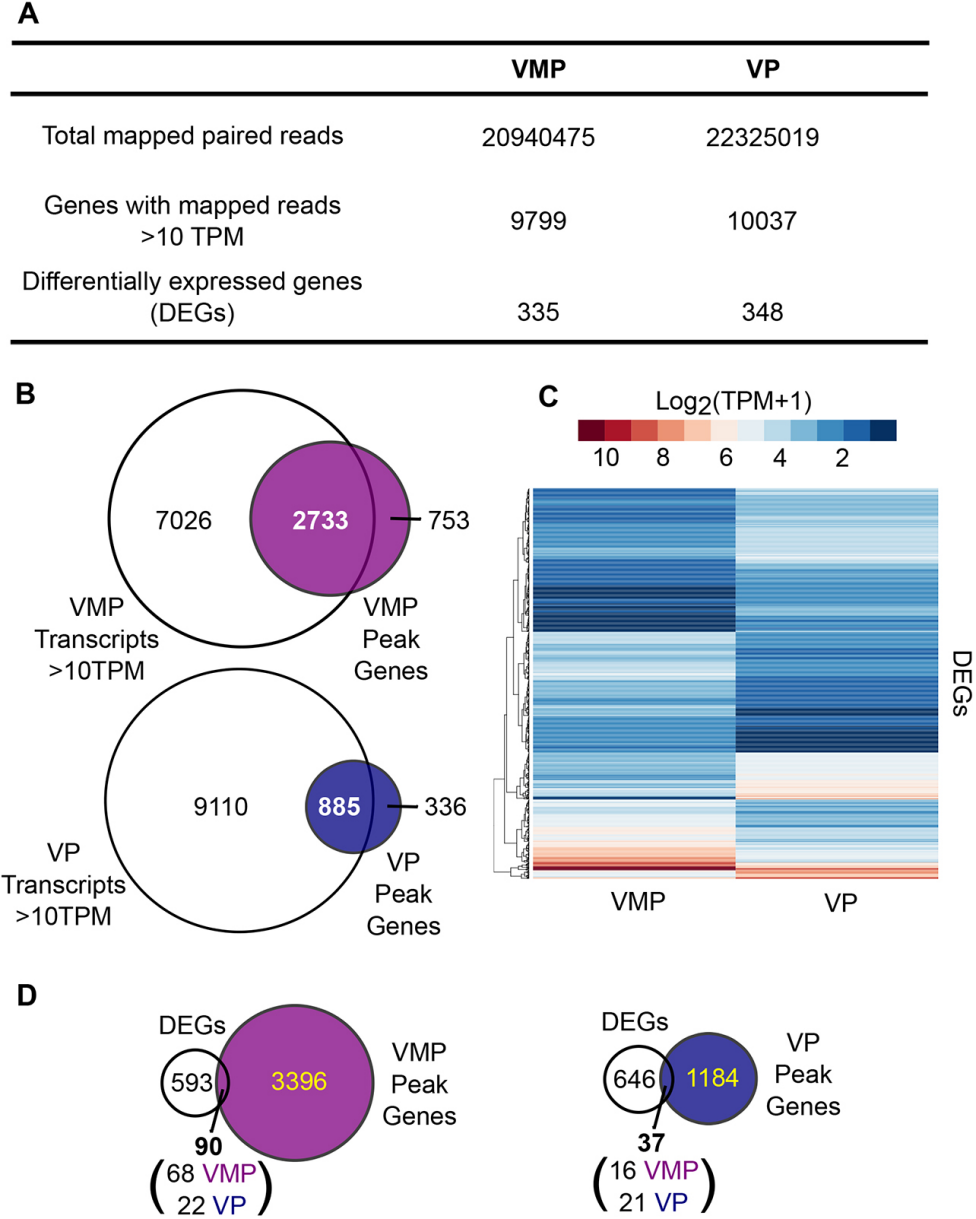
**Identification of VMP and VP whole tissue cistromes.** (A) Overview of RNA-seq transcriptomes of VMP and VP detailing the number of paired reads mapped to the rn6 genome, the number of genes with read counts >10 TPM and the number of DEGs between VMP and VP identified by NOISeq. (B) Transcript validation of genes associated with upstream AR peaks (between +100 and −100,000 bp upstream of the transcription start site) from VMP and VP (Peak Genes). Venn diagrams illustrating that 2733 (78%) and 885 (72%) genes with an upstream AR peak have read counts >10 TPM in VMP and VP transcriptomes, respectively. (C) Heatmap representing the log_2_ expression values (TPM+1) of the 683 DEGs identified by NOISeq between VMP and VP tissues. (D) Venn diagrams illustrating that 90 DEGs (68 VMP enriched and 22 VP enriched) overlap with genes associated with an upstream AR peak in VMP and 37 DEGs (16 VMP enriched and 21 VP enriched) overlap with genes with an upstream AR peak in VP.

Next, we performed transcriptional profiling of our male and female tissues in order to identify genes that were differentially regulated by androgens during prostate development; we then compared these to ARTGs derived by ChIP-seq. To perform a comparison using tissue with the closest developmental homology, as well as reducing cellular heterogeneity, we focussed our analysis on VMP and VP tissues. VMP is the anatomic female equivalent of the VP and is known to possess prostate inductive activity if exposed to testosterone (Thomson et al., 2002; Timms et al., 1995). For a global comparison of transcript data with AR ChIP-seq data, we identified VMP and VP transcripts with a read count >10 TPM (Fig. 3A) and compared these with VMP and VP ARTGs (genes with an AR peak within 100 kb 5′ of TSS by ChIP-seq). This defined VMP and VP AR cistromes. We observed 2733 VMP and 885 VP genes associated with an AR binding site; 72–78% of AR peaks were associated with an expressed transcript (Fig. 3B).

To identify which of these AR target transcripts were androgen regulated, we used NOISeq (Tarazona et al., 2011) to identify sexually dimorphic differentially expressed genes (DEGs) between VMP and VP. Results showed 335 VMP-enriched and 348 VP-enriched genes (Fig. 3A). These were visualized by heatmap, supporting their differential expression between VMP and VP (Fig. 3C). To determine whether these DEGs represented androgen-responsive genes during prostate development, we compared them with differentially expressed transcripts in response to testosterone in an embryonic mouse model of androgen-treated UGS (Schaeffer et al., 2008). This identified 107 common transcripts, validating our approach in identifying androgen-regulated transcripts (Fig. S5A). A smaller subset of our genes were also co-identified as differentially regulated during prostate organogenesis in mice (Pritchard et al., 2009) (Fig. S5B). Gene ontology analysis of the DEGs identified enrichment in pathways related to tube morphogenesis and development of branching structures, validating our ability to identify genes involved in developmental processes (Fig. S6). For a more targeted approach to identifying androgen-regulated ARTGs, we next compared the DEGs to VMP and VP ARTGs (Fig. 3D) and identified 90 DEGs associated with an AR peak in VMP (68 VMP-enriched genes and 22 VP-enriched genes) and 37 DEGs associated with an AR peak in VP (16 VMP-enriched genes and 21 VP-enriched genes). The proportion of AR peaks associated with DEGs was low: 13% in VMP and 5% in VP. This result is consistent with a model of indirect regulation of ARTGs, where primary targets of AR regulate a cascade of secondary or tertiary events. We examined whether the sexually dimorphic ARTGs were associated with actively transcribed genes in human *in vivo* by a comparison with the genome-wide transcriptome of human embryonic prostate tissue (Orr et al., 2012). We co-identified 48% (44) sexually dimorphic VMP ARTGs and 67% (25) of VP sexually dimorphic ARTGs in human embryonic prostate tissues, validating our ability to identify physiologically relevant transcripts (Fig. S7). A similar analysis was conducted combining female VMP and SU samples and combining male VP and DP samples to identify global differences in gene expression and ARTGs between females and males. This identified fewer DEGs between females and males than for VMP versus VP analysis (83 female enriched and 50 male enriched) of which 35 were associated with a female AR peak and 36 were associated with a male AR peak (Fig. S8).

### Identification of mesenchymal-specific sexually dimorphic AR target genes using scRNA-seq

VP is composed of both epithelium and mesenchyme, and in whole tissue RNA-seq analysis it is possible that DEGs between VMP and VP are derived from differences in tissue composition, such as the inclusion of epithelia. Furthermore, VMP and VP mesenchyme are composed of multiple cell subsets (Boufaied et al., 2017). In order to overcome epithelial cell contamination and focus on mesenchymal-specific AR targets, we performed scRNA-seq of dissociated VP and VMP mesenchyme. Tissue pools of microdissected VMP and VP tissues were dissociated into single cells using collagenase. Collagenase digestion dispersed the extracellular matrix, freeing mesenchymal cells from collagen but leaving epithelial branching structures intact. Cell straining removed the branched epithelial structures and isolated purified mesenchymal cells. Single cell suspensions of VMP and VP mesenchyme were run on a Fluidigm C1 chip system, RNA was extracted and scRNA-seq libraries prepared. To verify the mesenchymal identity of our cells, we examined the expression of a panel of known epithelial markers and stromal/mesenchymal markers in both VMP and VP scRNA-seq datasets. We confirmed an enrichment of stromal markers versus epithelial markers in both datasets, validating the identity of the cells as mesenchymal (Fig. S9).

**Fig. 4.**
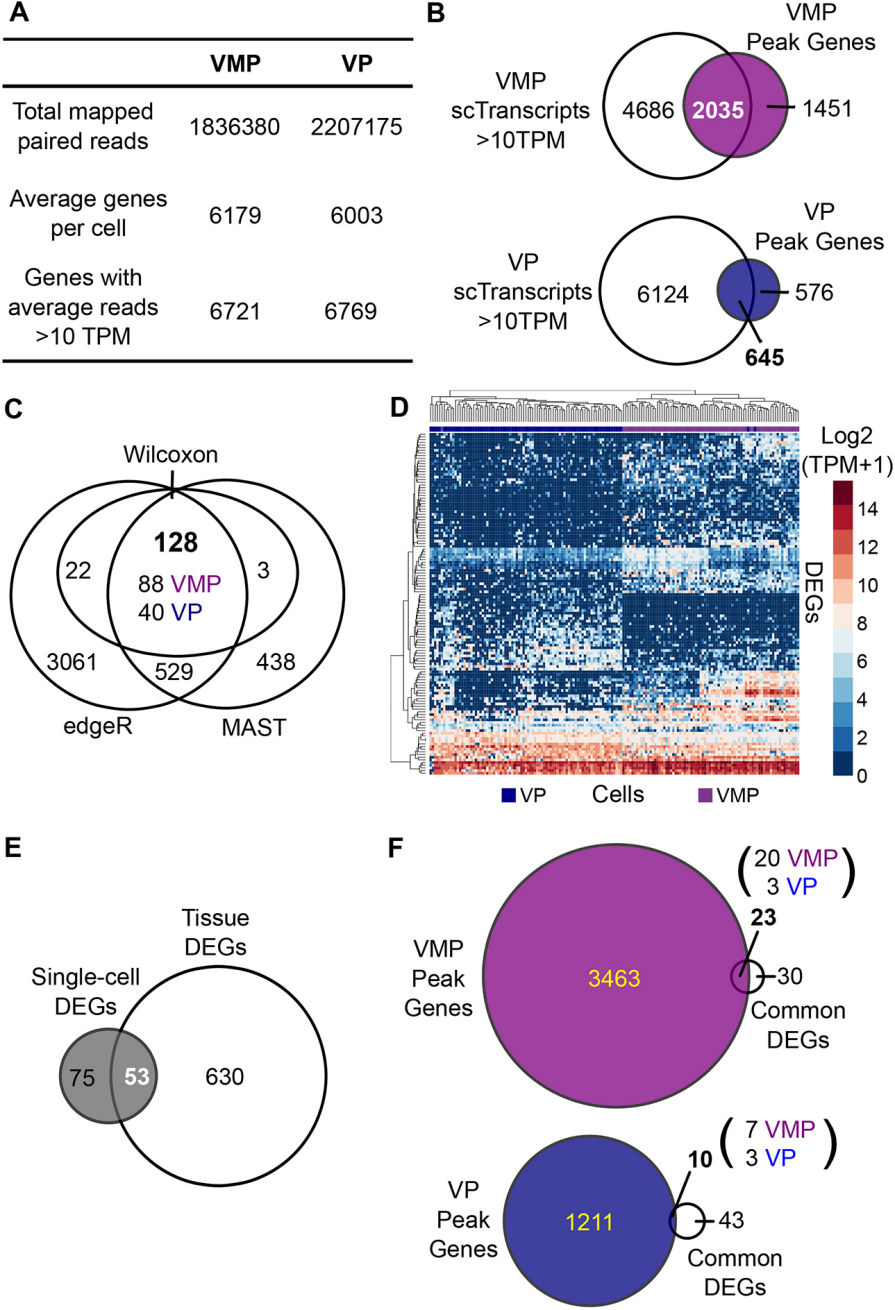
**Identification of VMP and VP single-cell cistromes.** (A) Overview of scRNA-seq transcriptomes of VMP and VP detailing the number of paired reads mapped to the rn6 genome, the average number of genes detected per cell and the genes with average read counts >10 TPM. (B) Transcript validation of genes associated with upstream AR peaks (between +100 and −100,000 bp upstream of the transcription start site) from VMP and VP (Peak Genes). Venn diagrams illustrating that 2035 (58%) and 645 (53%) genes with an upstream AR peak have average read counts >10 TPM in VMP and VP single cell transcriptomes, respectively. (C) DEGs were identified between VMP and VP single cells using three algorithms: edgeR Quasi-Likelihood *F*-test adjusting for cellular detection rate, MAST using cpms and adjusting for cellular detection rate and the non-parametric Wilcoxon test using Seurat. In all cases, genes were considered differentially expressed provided they had a false discovery rate (FDR) of <0.05 (edgeR and MAST) or a Benjamini–Hochberg adjusted *P*-value of <0.05 (Wilcoxon). Venn diagram illustrates the overlap between DEGs identified by the three methods. 128 genes were identified by all three methods, with 88 VMP enriched and 40 VP enriched. (D) Heatmap representing the expression values, log_2_ (TPM+1), of the 128 DEGs identified by edgeR, MAST and Wilcoxon between VMP and VP single cells. (E) Venn diagram of the 683 DEGs identified in whole tissue RNA-seq by NOISeq and the 128 identified by edgeR, MAST and Wilcoxon in scRNA-seq between VMP and VP. A total of 53 common genes were identified between tissue and scRNA-seq. (F) Venn diagrams illustrating that 23 common DEGs (20 VMP enriched and 3 VP enriched) overlap with genes associated with an upstream AR peak in VMP and 10 DEGs (7 VMP enriched and 3 VP enriched) overlap with genes with an upstream AR peak in VP.

Our scRNA-seq data were quality controlled using Scater (McCarthy et al., 2017) to remove cells with low library size and low number of mapped genes as well as a high ratio of mitochondrial DNA and spike-in controls. The distributions of these factors are shown in Fig. S10. The average read and gene statistics as well as the numbers of cells that passed quality control are shown in Table S2. In total, 94 VMP and 91 VP single cells from two replicate batches were combined and used for further analysis. The landscape of cells from the combined dataset in two-dimensional space was shown by principal component analysis and demonstrated a clear separation of VMP and VP cell types, confirming that tissue identity was retained in dissociated single cells (Fig. S11). Cell cycle differences were analysed and the proportion of cells in each cell cycle phase for all samples are shown in Fig. S12A. Data were examined for other technical confounding factors as well as cell cycle to detect unwanted variation within our data (Fig. S12B). Of these, only batch effects represented a median of >3% of gene expression variance, with other technical factors only having a minor confounding effect (Fig. S12C). We therefore regressed out batch effects in our downstream analyses. The average number of mapped reads, genes detected per cell and genes with read counts >10 TPM are shown in Fig. 4A.

In a global comparison of mesenchymal-specific transcripts from scRNA-seq with ChIP-seq ARTGs, we took genes with an average read count of >10 TPM in VMP and VP single cells and compared these with our VMP and VP ChIP-eq ARTGs. We identified 2035 VMP and 645 VP genes associated with a VMP or VP AR binding site, respectively (Fig. 4B).

To identify mesenchymal-specific androgen-regulated transcripts, we used three methods to identify DEGs in our single cell data. Using an edgeR quasi-likelihood *F*-test adjusting for cellular detection rate and batch effects yielded 3740 DEGs with an FDR value of <0.05. Using MAST with counts per million expression values and adjusting for cellular detection rate and batch effects yielded 1098 DEGs with an FDR value of <0.05. We also used a non-parametric Wilcoxon test following normalizing, filtering and scaling of combined VMP and VP single-cell data using Seurat (Satija et al., 2015). This yielded 128 DEGs with a Bonferroni–Hochberg adjusted *P*-value of <0.05. The performance and suitability of these methods were recently reviewed (Soneson and Robinson, 2018). We identified 128 transcripts common to all three methods, of which, 88 were VMP-enriched and 40 were VP-enriched transcripts (Fig. 4C). Visualization of the 128 DEGs by heatmap and hierarchical clustering showed a clear separation of VMP and VP single-cell populations (Fig. 4D). Gene ontology analysis of these identified enrichment in pathways related to urogenital system development, validating our ability to identify genes involved in developmental processes (Fig. S13). We identified 24% of our DEGs as androgen regulated in murine prostate development *in vivo* (Schaeffer et al., 2008) (Fig. S14A) whereas 2% were identified as markers of prostate organogenesis (Pritchard et al., 2009) (Fig. S14B). The lower degree of overlap compared with whole tissue RNA-seq derived DEGs probably reflects the underrepresentation of mesenchymal transcripts in these published microarray datasets (de Bruin et al., 2005). Next, we compared our sexually dimorphic DEGs from whole tissue RNA-seq with those from scRNA-seq to derive robust candidate genes identified by two independent transcriptomic methods. Fig. 4E shows a Venn diagram of DEGs from the whole tissue analysis (683) compared with DEGs from scRNA-seq (128), which identified 53 transcripts as common to both. The expression of these in both whole tissue and scRNA-seq data were visualized by heatmaps, violin plots and bar plots (Fig. S15).

To determine which of these androgen-regulated transcripts were also direct targets of the AR in mesenchyme, and to derive our final sexually dimorphic AR cistromes, we compared the 53 DEGs with both VMP and VP ARTGs which yielded 23 VMP and 10 VP genes associated with an AR binding site (ARBS), respectively (Fig. 4F). Comparison of these with the human embryonic transcriptome (Orr et al., 2012) showed a high degree of overlap (48–72%), suggesting that these are also expressed in developing human prostate (Fig. S16).

To assess the AR–DNA binding characteristics of these candidate genes, we evaluated the genomic distribution of ARBSs and observed a lower proportion of sites in intergenic regions in VMP (76%) than in VP (94%) (Fig. S17A). This is also reflected in the position of the ARBS relative to gene TSS, consistent with our previous observations using total ARBSs for the same samples, and suggests a shift of AR binding to intergenic regions upon stimulation with androgens. In addition, we performed *de novo* motif analysis on the AR cistrome peaks using HOMER. Surprisingly, we did not observe AR binding to classical AREs in either VMP or VP (Fig. S17B). However, because of the low number of starting AR peaks, we were unable to identify enriched DNA-binding motifs that were statistically significant. Using our top hits, despite low numbers, our analysis identified *Stat1* and *Elf3* as possible cofactors for AR in VMP and VP, respectively (Fig. S17B).

Although our motif analysis was inconclusive, we speculate that differential expression of sexually dimorphic genes between VMP and VP are not driven by AR binding to classical AREs.

### Validation of sexually dimorphic transcript expression and response to androgens by qPCR

**Fig. 5.**
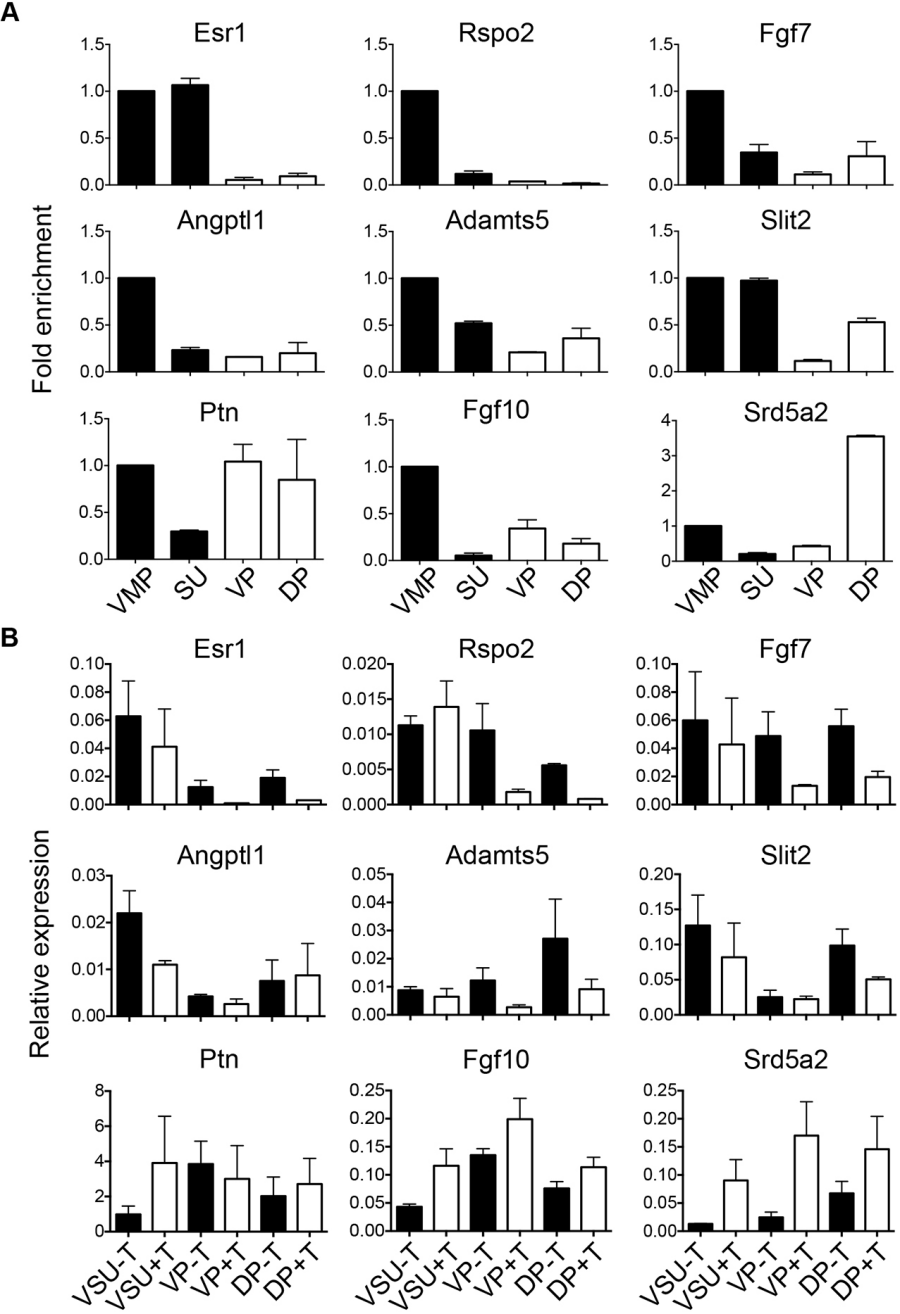
**Validation of dimorphism and androgen responsiveness of VMP and VP cistromes in female and male P0 rat whole tissues and *ex vivo* organ cultures.** (A) qPCR of whole VMP, SU, VP and DP tissues. The top row shows genes with a peak in VMP only, the middle row shows genes with a peak in both VMP and VP and the bottom row are control transcripts previously shown to be sexually dimorphic in rat developmental prostate. Elevated levels of VMP-enriched cistrome genes were observed versus male tissues (*Esr1*, *Rspo2*, *Fgf7*, *Angptl1*, *Adamts5* and *Slit2*). Control sexually dimorphic genes *Ptn* and *Srd5a2* were elevated in VP and DP male tissues compared with VMP and SU. *Fgf10* was enriched in VMP compared with all other tissues. Data are represented as mean fold difference relative to VMP (±s.d.) of duplicate biological replicate tissue pools (*n*=2). (B) qPCR of *ex vivo* organ cultures of whole female urogenital tract tissues (VSU; containing both VMP and SU), VP and DP tissues treated with 0.01 nM testosterone (+T) or ethanol (−T) for 24 h. Genes with AR peaks in VMP only are downregulated in response to testosterone in VP and DP organ cultures (*Esr1*, *Rspo2* and *Fgf7*). Genes with a peak in both VMP and VP show variable response to testosterone whereas *Fgf10* and *Srd5a2* control transcripts are upregulated in response to testosterone in organ cultures. Data are represented as relative to expression of four housekeeping genes (±s.d.) of duplicate biological replicates (*n*=2).

To validate whether our candidate genes were differentially expressed between males and females, we performed a Taqman qPCR array on pooled microdissected tissues for a subset of our AR cistrome genes that were common between our ChIP-seq, whole tissue RNA-seq and scRNA-seq analyses. We included VMP, SU, VP and DP tissues. We examined 12 candidates by qPCR as well as a panel of control genes showing dimorphic expression (*Ptn*, *Fgf10* and *Srd5a2*) and genes equally expressed in females and males (*Ebf3* and *Meis2*) (Fig. 5A and Fig. S18). Overall, our candidate genes showed sexually dimorphic expression, and the majority were VMP-enriched compared with male tissues. A subset of these have been previously reported as sexually dimorphic in urogenital tract tissues (Chrisman and Thomson, 2006; Mehta et al., 2011; Thomson and Cunha, 1999) validating our bio-informatic approach in identifying sexually dimorphic genes.

To verify whether our candidate genes were also androgen responsive, we performed *ex vivo* organ cultures of microdissected female urogenital tract tissues (containing both VMP and SU; VSU) and male microdissected VP and DP tissues. Organ cultures were treated with 10 nM testosterone or ethanol for 24 h. Overall, we observed a reduction in expression of our candidates in response to testosterone in VP samples, with variable response in female VSU tissues (Fig. 5B). We noted that genes with an AR peak in VMP only (*Esr1*, *Rspo2* and *Fgf7*) versus those that have a peak in both VMP and VP showed a more pronounced response to testosterone. *Ptn* and *Fgf10* showed variable response to testosterone whereas *Srd5a2* was upregulated in response to testosterone as expected, validating our experimental approach. These results identify new ARTGs, as defined by AR genomic binding, sexually dimorphic transcript expression and response to testosterone in organs *ex vivo*. Surprisingly, the majority of AR targets were repressed by androgens.

To assess whether our candidate AR-regulated genes are involved in human reproductive development, we investigated transcript expression in fibroblasts derived from patients with disorders of androgen action. We examined a panel of genes by Taqman qPCR in primary genital fibroblasts from 13 patients with complete androgen insensitivity syndrome (CAIS). Of these patients, nine had wild-type AR and four had mutated AR, as defined by exon sequencing. We observed high variability in the expression of our candidate genes, although both over- and underexpression of candidate genes was noted among different individuals (Fig. S19). Importantly, there were no candidate genes showing a consistent or similar pattern of misexpression across all patients.

### Analysis of cellular heterogeneity of AR and AR targets using scRNA-seq

**Fig. 6.**
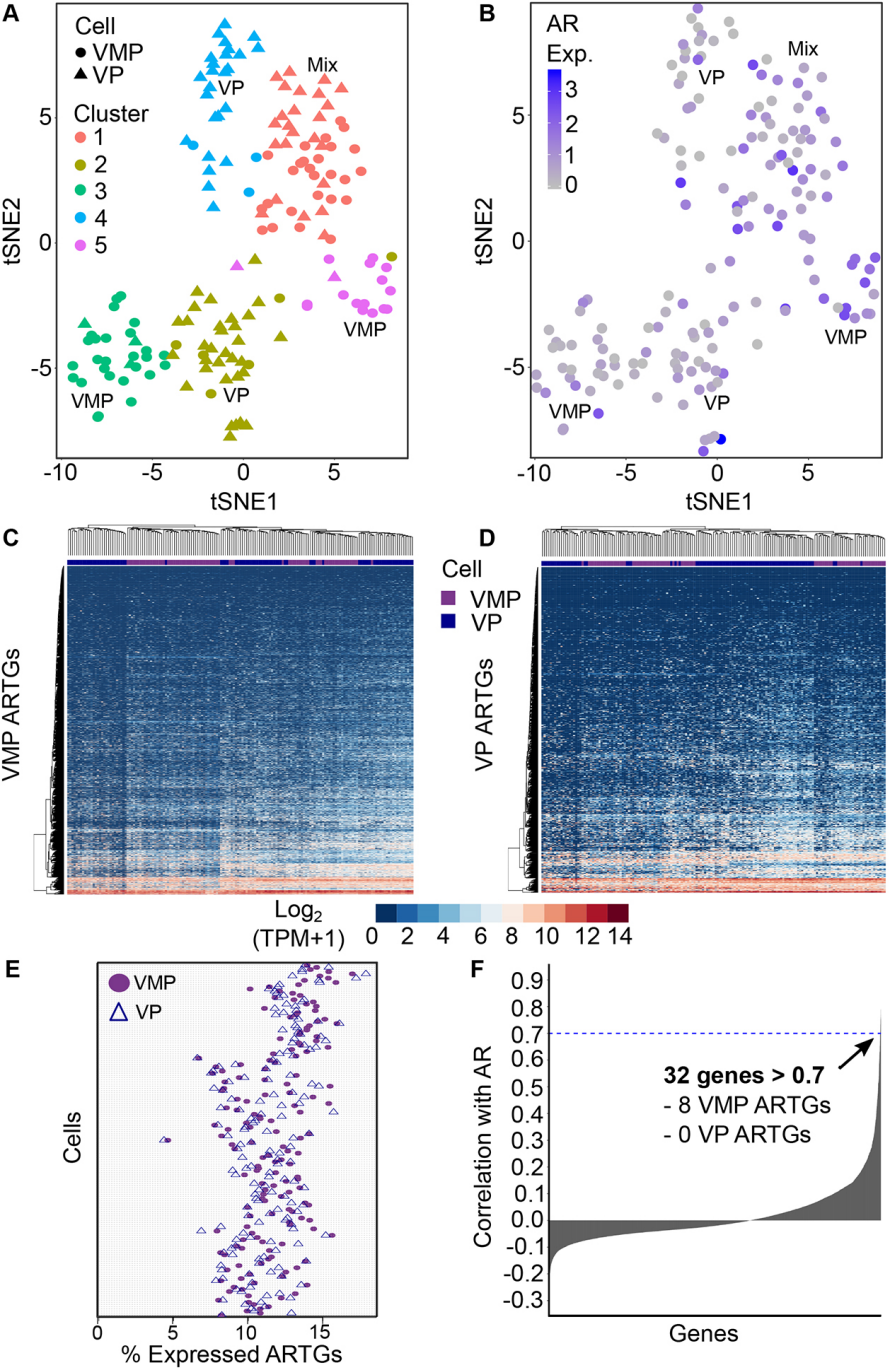
**Evaluation of cell heterogeneity within VMP and VP and characterization of AR and ARTG expression across cell subpopulations.** (A) tSNE analysis identified five cell subpopulations in the combined VMP/VP scRNA-seq dataset, which split into two VMP clusters, two VP clusters and one mixed VMP/VP cluster. (B) Visualization of AR expression across the five cell subpopulations. AR expression is presented as normalized, log-transformed and scaled expression relative to all other cells in the dataset. AR expression shows a random distribution across cells and is not associated with cell subpopulations. (C,D) Heatmaps representing the log_2_ expression values (TPM+1) of VMP ChIP-seq ARTGs (C) and VP ChIP-seq ARTGs (D) across cells. VMP and VP ARTGs were not sufficient to cluster cells into discrete subpopulations. (E) Dot chart representing the percentage of VMP ARTGs (purple circles) and VP ARTGs (blue triangles) with a positive relative expression value in normalized, log-transformed and scaled scRNA-seq data. The percentage of expressed ARTGs is stable across the single cell populations, suggesting that ARTGs are not enriched in specific cell populations. (F) Waterfall plot showing the Pearson correlation values for the expression of each gene in the scRNA-seq transcriptome versus AR expression. Only 32 genes had a correlation value >0.7 (blue dashed line). Of these, 8 were VMP ARTGs and 0 were VP ARTGs.

Our results had identified a group of candidate mesenchyme-specific ARTGs, and our next goal was to determine whether these were expressed uniformly throughout mesenchymal cells or within mesenchymal subsets. First, we used Seurat to assess the degree of cellular heterogeneity and subset composition in VMP and VP single-cell datasets. A previous study showed that the VMP is comprised of two subpopulations (Boufaied et al., 2017). Consistent with this finding, we observed that the VMP was not homogeneous and was divided into subpopulations, using a combined dataset with VP single cells. The subgroups were composed of two VMP cell clusters, two VP cell clusters and one cluster containing a mix of VMP and VP cells (Fig. 6A). We further characterized these subsets by identifying 310 transcripts that discriminated each of the five clusters using Seurat and a non-parametric Wilcoxon statistical test. Expression of the top markers for each cluster was visualized by heatmap and violin plots (Fig. S20). Comparison of these published markers of human and mouse adult prostate stromal cell populations co-identified genes associated with adult smooth muscle cell populations; overall, our markers of developmental mesenchyme were poorly represented in adult prostate stroma (Fig. S21).

Next, we investigated whether AR and ARTGs were expressed throughout the mesenchyme uniformly, or if androgen-responsive subpopulations could be defined. Our goal was to determine whether AR clustered within a population, and whether ARTGs were expressed within the same population or distributed among other subgroups. We observed that AR expression was not enriched in specific subpopulations of cells and was randomly distributed across all cells (Fig. 6B). Visualization of our VMP and VP ARTGs by heatmap and hierarchical clustering analysis showed that our ARTGs did not cluster cells into discrete cell subpopulations (Fig. 6C,D). In addition, we determined that the percentage of ARTGs expressed in individual cells was consistent, between 5–17% across the population. This suggested that our ARTGs were not enriched in specific cells or cell subpopulations (Fig. 6E) but were partially expressed across all cells in the population. To determine whether AR expression was associated with increased or decreased expression of any genes in the scRNA-seq transcriptome, we performed a Pearson correlation analysis of AR expression versus expression of each individual gene of the transcriptome. We found that a surprisingly low number of genes were correlated with AR expression (32 with a correlation of >0.7). Comparison of these with our ChIP-seq ARTGs identified eight VMP ARTGs and zero VP ARTGs in common (Fig. 6F). Overall, our results suggest that neither AR nor its target genes were associated with specific cell subpopulations and that the expression of AR was not strongly correlated with the expression of other genes in the transcriptome. This was confirmed by analysis of VMP and VP cells separately (Fig. S22).

## DISCUSSION

The role of androgens and AR signalling in sexually dimorphic development of reproductive tissues is well established, as is the requirement for AR signalling in mesenchymal cells (Cunha and Lung, 1978; Donjacour and Cunha, 1993). This led to the hypothesis that AR action in mesenchyme leads to expression of genes that regulate epithelial proliferation and differentiation, such as paracrine acting factors or andromedins (reviewed in Tenniswood, 1986; Thomson, 2008; Toivanen and Shen, 2017). The mesenchymal compartment is composed of different subsets defined morphologically and functionally (Marker et al., 2003; Timms et al., 1995) as well as at the molecular level (Boufaied et al., 2017; Vanpoucke et al., 2007). The urethral smooth muscle layer is another stromal subset that may play a role in AR action during prostate development (Chrisman and Thomson, 2006; Thomson et al., 2002). The function of AR within these subsets has not been investigated directly, despite the known functional importance of AR signalling within this compartment. Here, we performed an in-depth analysis of AR using genome-wide AR binding and transcriptomic analysis of male and female urogenital mesenchyme to document the molecular events during sexually dimorphic development of the prostate.

We observed distinct differences in AR genomic binding characteristics between female and male tissues. Enrichment of the AR at proximal promoter regions of genes was found in female tissues, whereas AR was enriched at intergenic regions in male tissues. The enrichment of ARBSs at gene promoters coupled with the lower levels of testosterone in females could be indicative of androgen-independent AR signalling (Decker et al., 2012) or a characteristic feature of stromal AR binding (Nash et al., 2017). Proximal promoter ARBSs have recently been observed in some tumour samples; however, they may be derived from stromal cells within these samples (Pomerantz et al., 2015). We cannot exclude the possibility that the higher proportion of ARBSs at intergenic regions in male tissues reflects an epithelial AR binding profile, which could explain the enrichment of AR downstream of the adult prostate epithelial AR target *Zbtb16*. However, we showed that the majority of AR protein expression is mesenchymal in origin at the developmental stage used in our analysis and took steps to exclude possible epithelial AR targets in our downstream experiments and analyses. Further epigenetic profiling studies of these tissues are required to define the function of AR at these genomic regions, but we propose that these may be defining features of AR action between males and females. We also observed differences in the DNA sequences to which AR binds in females versus males. Female AR binding sites were enriched for sequences associated with members of the nuclear factor I (NFI) family of transcription factors, whereas male AR binding sites were enriched for the classical palindromic androgen response elements (AREs). This suggests that NFI transcription factors act as co-factors for the AR in females. NFI factors and AR have been shown to co-regulate known ARTGs such as PSA and FKBP5 in prostate cancer cell lines (Grabowska et al., 2014, 2016) and could play a role here in regulating organ development (Campbell et al., 2008; Gründer et al., 2002; Messina et al., 2010; Murtagh et al., 2003; Steele-Perkins et al., 2005). However, further experimental work is required to show direct interactions between AR and NFI family members in our tissues.

We propose that a feature of androgen-driven dimorphism of the prostate is the reprogramming of AR binding from gene promoter regions to distal enhancer sites containing AREs, which has been reported in prostate cancer epithelial cells (Decker et al., 2012; Massie et al., 2011; Sharma et al., 2013) as well as in response to androgens in prostate cancer associated fibroblasts (Cioni et al., 2018).

We are the first to assess sexually dimorphic gene expression in mesenchymal cells within prostate tissue using both RNA-seq and scRNA-seq. Our transcriptomic analysis did not identify a strong bias towards male-enriched genes as would be expected under the classical andromedin hypothesis (reviewed in Tenniswood, 1986; Thomson, 2008). We observed that a significant number of molecules were repressed by androgen signalling, in contrast to those upregulated, and this pattern was similar in our validation studies where there was a general trend towards repression of transcript expression. The underlying molecular mechanisms of AR-mediated gene repression are poorly understood but is probably achieved through a delicate balance of co-repressors and chromatin remodelling complexes (reviewed in Grosse et al., 2012). Indeed, NFIC, a potential co-factor found in our ChIP-seq analysis in female tissues, has been found to function as a co-repressor of ARTGs in prostate cancer epithelial cells (Grabowska et al., 2014). We speculate that a key function of testosterone in addition to activation of male-specific ARTGs is to repress female-specific genes. Nevertheless, we identified several genes previously shown to be both up- and downregulated by androgen and required for prostate development (Pritchard et al., 2009; Schaeffer et al., 2008; Thomson et al., 1997), which validates our approach.

By comparing DEGs to ARTGs identified by ChIP-seq we defined a relatively small number of sexually dimorphic ARTGs. This suggests that a large proportion of molecular determinants of prostate masculinization are either not direct targets of the AR (and indirectly driven by androgen signalling) or regulated by AR bound at distal enhancers that we were unable to characterize using ChIP-seq. Further experimentation incorporating techniques such as DNase sequencing (Song and Crawford, 2010), ATAC sequencing (Buenrostro et al., 2015), ChIA-PET (Fullwood et al., 2009) and Hi-C (Lieberman-Aiden et al., 2009) would be required to address this. However, our analysis did identify genes such as *Esr1* and *Rspo2*, which have previously been reported as sexually dimorphic during prostate development. Mesenchymal expression of *Esr1* is important for prostate branching morphogenesis (Chen et al., 2009; Chen et al., 2012) and is testosterone responsive (Chrisman and Thomson, 2006). *Rspo2* is a key regulator of the Wnt signalling pathway (Nam et al., 2006), is expressed in murine urogenital mesenchyme (Mehta et al., 2011) and is androgen responsive during murine prostate development (Schaeffer et al., 2008). In addition, Rspo family member upregulation is a feature of sexual differentiation of murine gonadal cells (Harris et al., 2018). This, coupled with the differential regulation of several of our candidate genes in response to testosterone treatment in our *ex vivo* organ culture model validates our approach to identifying androgen-driven ARTGs. We are the first to identify ARBSs proximal to *Esr1* and *Rspo2* transcriptional start sites in urogenital mesenchyme and propose that these are direct ARTGs, which are androgen repressed during prostate development. Our analysis also identified *Fgf7* as an androgen-repressed ARTG, despite other studies proposing FGF-7 as a candidate andromedin (Sugimura et al., 1996; Yan et al., 1992). Interestingly, *Fgf7* mRNA was shown to decrease in response to testosterone (Fig. 5), concordant with previous studies using prostate organs grown *in vitro* (Thomson et al., 1997). The analysis of our candidate ARTGs in patients with CAIS showed that there was significant variability among transcript expression levels, but that there were no ARTGs that showed a consistent pattern across all patients. This is important because it suggests that AR may regulate several targets, each of which may be required for masculinization in humans, and that there is no single gene common to all patients. This result is also consistent with a previous study, using fibroblasts derived from CAIS/PAIS patients, which observed limited concordance between individuals. Our studies confirm and extend this finding (Holterhus et al., 2007, 2003). The precise role of our candidate genes during prostate organogenesis is yet to be defined using ablation studies *in vivo* but provides a unique starting point for defining those required for dimorphic development of sex-accessory tissues.

We are the first to perform both AR ChIP-seq and scRNA-seq on mesenchymal tissues, allowing the characterization of AR signalling and ARTGs distribution across single cells and cell subpopulations. Our scRNA-seq analysis revealed limited heterogeneity within VMP and VP mesenchymal cells, in contrast to studies conducted using adult prostate stroma (Henry et al., 2018; Kwon et al., 2019). Our mesenchymal cell subpopulations were largely distinct from those found in normal human and rodent adult prostate stroma (Fig. S21) (Henry et al., 2018; Kwon et al., 2019). This suggests that our mesenchymal cell subpopulations are transient and that prostate stroma undergoes further differentiation prior to adulthood. However, these comparisons are confounded by the limited number of single cells analysed in our study and by cross-species differences. A time course experiment assessing the changes in stromal cell populations at different developmental stages would be required to address this question but was beyond the scope of this study. Investigation into how our ChIP-seq ARTGs distribute across these cell populations suggests that AR signalling targets are not driving cellular heterogeneity; because cells did not cluster cells based on ARTGs. Similarly, the mRNA levels of the AR itself were randomly distributed across cell populations, suggesting that neither AR nor its target genes are markers of cell subpopulations. Surprisingly, we were unable to derive other ARTGs by comparing AR-high versus AR-low cells, nor did we identify many genes that correlated with AR mRNA expression. It is well accepted that mRNA levels do not always reflect protein levels because of downstream translational control mechanisms (Liu et al., 2016). It is possible that AR protein abundance at the single cell level has a greater influence on the distribution and expression of ARTGs but this would need to be verified using advanced techniques such as single-cell proteomics (Su et al., 2017).

Overall, our study is the first to perform an in-depth AR genomic and transcriptomic analysis of mesenchymal tissues of the developing prostate. We document the differences in AR genomic binding profiles between males and females and the transcriptomic features of androgen-driven masculinization of the prostate. We are the first to combine AR ChIP-seq with scRNA-seq to further our understanding of how AR functions at the single-cell level and have found that AR and its target genes transcend cellular identity and heterogeneity. We suggest that further verification of AR binding patterns and how these relate to transcription of sexually dimorphic genes will eventually lead to a more complete understanding of how AR drives masculinization.

## MATERIALS AND METHODS

### Animal and tissue collection

Animal studies were approved by the McGill University Facility Animal Care Committee (FACC) and performed as per MUHC animal protocol number 2015-7670. Wistar rats were maintained under a 12-h light/dark cycle with a standard laboratory diet. P0 pups were sacrificed by cervical dislocation and decapitation. Urogenital tracts were extracted from both females and males and were microdissected and pooled to produce female ventral mesenchymal pad (VMP) and smooth muscle and urethra (SU) and male ventral prostate (VP) and dorsal/dorsolateral prostate (DP) tissue components with a Leica MZ6 dissection microscope. Testes from males and brain tissue from males and females were pooled to serve as western blot controls.

### Cell culture

LNCaP prostate tumour cells and immortalized human prostatic stromal cells overexpressing wild-type androgen receptor (WPMY1-AR) (Tanner et al., 2011) were maintained according to the literature (Nash et al., 2017). Primary genital fibroblasts were derived from CAIS patients, as described previously (Gottlieb et al., 1996). Primary fibroblasts were maintained in DMEM supplemented with 10% FBS and used at passages between 3 and 6. All primary cells were used under ethical approval 15-631-MUHC.

### Western blotting and immunohistochemistry

AR western blotting and AR immunohistochemistry of rat P0 tissues were performed as described previously (Nash et al., 2017).

### ChIP sequencing

ChIP, ChIP-qPCR and Illumina ChIP-seq library preparation was carried out by Active Motif (Carlsbad, CA, USA) as described previously (Nash et al., 2017). Reads were aligned to the rat genome (rnor6.0) using the Bowtie2 algorithm with default settings (Langmead and Salzberg, 2012). AR peak data were processed as described previously (Nash et al., 2017). Visualization of ChIP-seq read coverage was performed using IGB software (Nicol et al., 2009).

### *Ex vivo* organ culture and testosterone treatment

Ventral prostate, dorsal prostate and female urethra (VSU; VMP, smooth muscle, urethra) were microdissected from P0 rat pups and placed in serum-free organ culture (Thomson et al., 1997). Organs were left in culture overnight, followed by treatment with testosterone (1×10^−8^ M) or vehicle for 48 h before harvesting for RNA isolation.

### RNA extraction and TaqMan^®^ qPCR array analysis

Total RNA was extracted from pooled tissues and *ex vivo* organ cultures using Qiazol followed by the RNeasy™ Mini kit (Qiagen, Venlo, Netherlands) following the manufacturer's instructions. Complementary DNA synthesis was performed using the High Capacity cDNA Reverse Transcription kit (Applied Biosystems-ThermoFisher Scientific, MA, USA) according to the manufacturer's instructions. Duplicate biological samples were analysed using a custom TaqMan^®^ qPCR array as per manufacturer's instructions on an ABI 7500 Fast machine (ThermoFisher Scientific, MA, USA). Transcript abundance was normalized to four housekeeping genes: *Gapdh*, *Tbp*, *Gusb* and *Mt-atp6*.

### RNA sequencing library preparation, data processing and differential gene expression

RNA sequencing was carried out by Exiqon, Inc. (Denmark) as described previously (Nash et al., 2017). Sequencing reads were aligned to the rat genome (rnor6.0) using the Tophat 2.1.0 algorithm (Kim et al., 2013) and only uniquely mapped and non-redundant genes were used for further analysis. Read counts were quantified using summarizeOverlaps from the GenomicAlignments R package (Lawrence et al., 2013). Transcripts with a read count of zero in all samples were removed. EdgeR (Robinson et al., 2010) was used for TMM normalization of reads and only transcripts with >1 counts per million were used to determine DEGs. The NOISeq R package (Tarazona et al., 2011) was used to screen DEGs between female (VMP and SU combined) and male (VP and DP combined) as well as between VMP and VP tissues. Genes with a *q*-value of ≥0.9 were considered differentially expressed. Comparisons of RNA-seq transcriptomes and DEGs with ChIP-seq data and their visualization were performed using R packages VennDiagram (Chen and Boutros, 2011) and NMF (Gaujoux and Seoighe, 2010), respectively.

### Single-cell RNA sequencing library preparation, data processing and differential gene expression

Single-cell dissociation, RNA extraction, library preparation, RNA-seq, read alignment and read quantification was performed on VMP and VP dissociated cells as described (Boufaied et al., 2017) using the Fluidigm C1 platform. Two biological replicate experiments were performed and combined for data analysis, annotating for batch.

The Scater R package (McCarthy et al., 2017) was used for quality control and normalization of scRNA-seq read count data. Low-quality cells were removed based on library size, number of genes detected, proportion of reads mapped to the mitochondrial genome and the ratio of reads to spike-in controls. Cells were removed if they met any of the following criteria: a median absolute deviation (MAD) value of <3 for library size, or number of mapped genes and a MAD value of >3 for ratio of reads mapped to mitochondrial DNA or to spike-in controls. The number of cells meeting these criteria are detailed in Table S2.

Differential gene expression was determined on a combined VMP and VP dataset between VMP and VP single cells using three different methods. EdgeR (Robinson et al., 2010) was used by incorporating cellular detection rate with a quasi-likelihood *F*-test. MAST (Finak et al., 2015) was used by incorporating cellular detection rate and using counts per million as described (Soneson and Robinson, 2018). For both algorithms, only genes with an estimated expression of >1 TPM in more than 25% of the cells were considered (Soneson and Robinson, 2018). For both algorithms, cellular detection rate and batch were passed as covariates for the analysis. Genes were considered significantly differentially expressed if they had a false discovery rate (FDR) of <0.05. A non-parametric Wilcoxon test was also used to identify DEGs using the Seurat R package (Satija et al., 2015). For this analysis, cells were filtered out if the number of detected genes was less than 3000 or more than 9500, or if the percentage of mitochondrial reads was greater than 30%. Read count data were normalized and log transformed using total gene expression; data were scaled while regressing out batch effects, percentage of mitochondrial reads and cellular detection rate using NormalizeData and ScaleData Seurat functions. Differential expression was tested using the FindMarkers Seurat function with default settings. Genes were considered significantly differentially expressed if they had a Bonferroni–Hochberg adjusted *P*-value of <0.05.

Comparisons of single-cell transcriptomes and DEGs to ChIP-seq data and their visualization were performed using R packages VennDiagram (Chen and Boutros, 2011) and NMF (Gaujoux and Seoighe, 2010), respectively. Clustering of single cell data was performed using Seurat and Pearson correlation analysis of AR expression with transcriptome genes using the corrr R package (https://CRAN.R-project.org/package=corrr).

### Gene ontology enrichment analysis

Gene ontology enrichment analysis was conducted using the clusterProfiler R package (Yu et al., 2012) on female versus male and VMP versus VP DEGs. Ontology terms with an FDR of <0.05 were deemed significant.
